# Preliminary data concerning the reliability and psychometric properties of the Greek translation of the 20-item Subjective Well-Being Under Neuroleptic Treatment Scale (SWN-20)

**DOI:** 10.1186/1744-859X-8-3

**Published:** 2009-01-21

**Authors:** Melina Siamouli, Katerina Moutou, Eleonora Pantoula, Stamatia Magiria, Irini Chatzivasileiou, Konstantinos Arapidis, Achileas Chatzivasileiou, Simeon Deres, Konstantinos N Fountoulakis

**Affiliations:** 1 Asclepius Mental Clinic, Veroia, Greece; 2 School of Medicine, Aristotle University of Thessaloniki, Greece; 3 State Mental Hospital of Thessaloniki, Greece; 4 Therapeutirio Spinari Mental Clinic, Kozani, Greece; 5 Agios Georgios Mental Clinic, Panorama Thessaloniki, Greece; 6 Third Department of Psychiatry, School of Medicine, Aristotle University of Thessaloniki, Greece

## Abstract

**Background:**

The 20-item Subjective Well-Being Under Neuroleptic Treatment Scale (SWN-20) is a self-report scale developed in order to assess the well-being of patients receiving antipsychotic medication independent of the improvement in their psychotic symptoms. The current study reports on the reliability and the psychometric properties of the Greek translation of the SWN-20.

**Methods:**

A total of 100 inpatients or outpatients with schizophrenia (79 males and 21 females, aged 42.6 ± 11.35 years old) from 3 different facilities were assessed with the Positive and Negative Symptoms Scale (PANSS), the Calgary Depression Scale and the Simpson-Angus Scale, and completed the SWN-20. The statistical analysis included the calculation of Pearson product moment correlation coefficient, the Cronbach α and factor analysis with Varimax normalised rotation.

**Results:**

The SWN-20 had an α value equal to 0.79 and all the items were equal. The factor analysis revealed the presence of seven factors explaining 66% of total variance. The correlation matrix revealed a moderate relationship of the SWN-20 and its factors with the PANSS-Negative (PANSS-N), PANSS-General Psychopathology (PANSS-G), the Simpson-Angus and the Calgary scales, and no relationship to age, education and income class.

**Discussion:**

The Greek translation of the SWN-20 is reliable, with psychometric properties close to the original scale.

## Background

In the past, the standard approach to the assessment of antipsychotic treatment was the rating of symptoms. In this frame, the patients' perspective concerning pharmacological treatment was largely neglected. However during the last couple of decades, especially after the development of second-generation antipsychotics (SGAs), research interest on this issue has markedly increased. Moreover, the interest concerning the quality of life of mental patients and their subjective sense of well-being, particularly of patients with schizophrenia has also increased. This increase is, at least partially, attributed to a supposed favourable effect of SGAs [1].

Quality of life (QoL) and subjective well-being (SWB) are different concepts with SWB being part of overall QoL. They both constitute a conceptual extension of therapeutic outcome criteria [2]. The patient satisfaction seems to correlate strongly to the patient's willingness to be or stay under any kind of treatment, thus determining the overall outcome to a significant extent [3]. In this context, the goals of treatment in schizophrenia nowadays include patient-related factors such as subjective response and quality of life. The patient's satisfaction with antipsychotic therapy is influenced by a number of different and maybe loosely related factors. These factors include medication adverse effects, psychoeducation, lack of involvement in decision making (concerning both the patient and his/her family) and the existence or absence of a therapeutic alliance [4,5]. However, the patient's perceptions concerning their treatment are not strongly related to the severity of illness or symptoms; on the contrary, there seems to be an association between perceptions of treatment and medication adverse effects. In essence, this means that the patient's perspectives markedly differ from these of his/her psychiatrist [6]. If this is the case, then a relative lack of adverse effects with newer drugs could result in higher levels of satisfaction and subjective well-being, but this remains to be proven [3,6,7]. The existence of both external and inner motivations underpinning the patients' attitudes towards medication and treatment in general suggests that any intervention needs to take into account both the disease and the person it afflicts, including his subjective experience, in a personalised way of treatment [8].

Methodologically, QoL and SWB are assessed with the use of rating scales, some of them being self-report ones. Although there are significant problems with definitions and with the reliability and validity of these scales, they constitute valuable tools for the assessment of the overall course of patients. Several studies have shown that the majority of schizophrenic patients are able to complete a self-rating scale in a reliable way [6].

The 20-item Subjective Well-Being Under Neuroleptic Treatment Scale (SWN-20) [9,10] is a self-report scale developed in order to assess the well-being of patients receiving antipsychotic medication, regardless of the improvement in their psychotic symptoms. The current study reports on the reliability and the psychometric properties of the Greek translation of the SWN-20.

## Methods

### Study sample

The study sample included 100 in or outpatients suffering from schizophrenia (21 females (21%) and 79 males (79%)) aged 42.6 ± 11.35 (range 19 to 65 years old). Participants came from three different private care facilities.

All patients gave informed consent and the protocol received approval by the Aristotle University of Thessaloniki's Ethics Committee.

### Clinical diagnosis

Diagnosis was made according to Diagnostic and Statistical Manual of Mental Disorders version IV Text Revision (DSM-IV-TR) criteria on the basis of a semi-structured interview. Patients were physically healthy with normal clinical and laboratory findings.

### Translation and back translation

**Figure 1:**
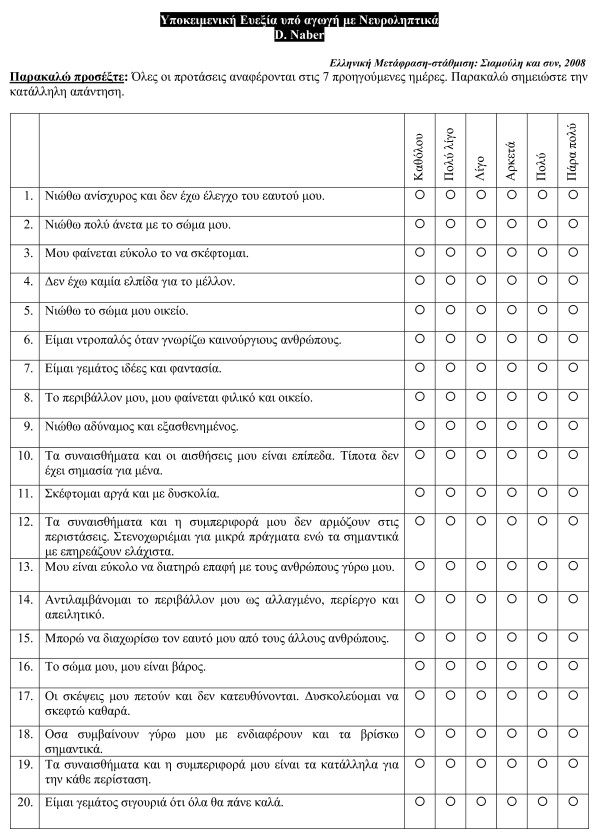
**Subjective Well-being Under Neuroleptic Treatment Scale (SWN) in Greek**.

Translation and back translation were made by two of the authors; one of whom did the translation and the other who did not know the original English text did the back translation. The final translation was finalised by consensus between them. The translated scale is shown in Figure 1.

### Psychometric assessment

All patients were assessed with the Positive and Negative Symptoms Scale (PANSS) (for the overall assessment of the severity of psychotic symptoms), the Calgary Depression Scale (for the assessment of depression), the Simpson-Angus Scale (for the assessment of side effects) and completed the SWN-20.

### Statistical analysis

Descriptive statistics for all scales were calculated. Item analysis [11] was performed, and the value of the Cronbach α for the SWN-20 was calculated. Principal component analysis (without and after Varimax normalised rotation) [12] was performed, and factor coefficients and scores were calculated. The Pearson correlation coefficient (R) was calculated to assess the relationship of the SWN-20 and its factors to the other psychometric tests.

## Results

The means and standard deviations for the scores for all scales are shown in Table 1. The SWN-20 had a Cronbach α equal to 0.79 and all the items were equal. The factor analysis (Table 2) revealed the presence of seven factors explaining 66% of total variance.

**Table 1:** Descriptive statistics of scales scores

**Scale**	**Mean**	**Minimum**	**Maximum**	**SD**
SWN-20	66.73	21	95	14.33

PANSS-Positive subscale	15.12	8	32	4.76

PANSS-Negative subscale	17.82	9	37	5.13

PANSS-General Psychopathology subscale	27.21	17	45	6.04

Calgary Depression Scale	1.27	0	11	2.20

Simpson-Angus Scale	1.59	0	8	2.14

SWN-20 factor 1	12.53	3	20	3.65

SWN-20 factor 2	16.22	2	25	5.29

SWN-20 factor 3	8.01	0	10	2.53

SWN-20 factor 4	13.34	1	20	4.16

SWN-20 factor 5	9.62	0	15	3.19

SWN-20 factor 6	16.71	1	25	5.31

SWN-20 factor 7	12.80	2	20	4.49

PANSS:Positive and Negative Symptoms Scale; SD: standard deviation; SWN-20:, Subjective Well-being under Neuroleptic Treatment scale.

**Table 2:** Results of the factor analysis of SWN-20

	**Factor 1**	**Factor 2**	**Factor 3**	**Factor 4**	**Factor 5**	**Factor 6**	**Factor 7**
SWN-1	**0.60**	0.30	0.12	0.25	-0.10	-0.05	0.18

SWN-2	0.02	**0.56**	-0.02	0.40	0.12	-0.02	0.19

SWN-3	-0.36	**0.52**	0.10	0.37	0.04	0.23	-0.22

SWN-4	-0.06	0.12	**0.76**	0.15	-0.02	-0.31	-0.09

SWN-5	0.00	0.27	0.36	0.22	**0.60**	0.06	0.12

SWN-6	-0.12	0.04	-0.02	-0.07	0.02	**0.77**	0.05

SWN-7	**-0.79**	0.16	-0.08	0.16	-0.02	-0.01	-0.01

SWN-8	0.09	-0.03	0.04	0.31	0.28	0.01	**0.70**

SWN-9	**0.30**	**0.44**	0.03	-0.07	-0.18	**0.36**	**0.39**

SWN-10	0.05	0.14	**0.70**	0.13	0.04	0.19	0.34

SWN-11	**0.44**	0.42	0.02	0.16	0.23	**0.56**	-0.06

SWN-12	0.22	-0.01	**0.80**	-0.12	0.14	0.15	0.02

SWN-13	-0.21	-0.16	0.28	**0.55**	0.12	**0.41**	**0.37**

SWN-14	0.06	0.30	0.11	-0.06	-0.05	0.10	**0.76**

SWN-15	-0.14	0.06	0.09	0.02	**0.75**	0.12	-0.14

SWN-16	-0.02	**0.73**	0.22	-0.13	0.28	0.10	0.19

SWN-17	0.19	**0.45**	0.12	**0.31**	0.02	**0.54**	0.13

SWN-18	0.01	0.04	-0.07	**0.81**	0.16	0.10	-0.06

SWN-19	0.14	0.06	-0.11	0.15	**0.76**	-0.10	0.32

SWN-20	0.02	0.22	0.22	**0.62**	0.04	-0.26	0.25

Proportion of total	8%	10%	10%	11%	9%	9%	9%

Total explained							66%

Values in bold underlined are factor loadings determining to which factor the specific item belongs to. SWN, Subjective Well-Being Under Neuroleptic Treatment Scale.

Some items load equally to more than one factor. Item 9 equally loads to factors 1, 2, 6 and 9. Item 11 loads to factors 1 and 6, item 13 to factors 4, 6 and 7 and item 17 to factors 2, 4 and 6.

Factor 1 includes items 1, 7, 9 and 11 and largely reflects 'mental control'. Factor 2 includes items 2, 3, 9, 16 and 17 and largely reflects a 'combined mental-physical control'. Factor 3 includes items 4, 10 and 12 and largely reflects 'depression and loss of emotional control'. Factor 4 includes items 13, 17, 18 and 20 and reflects 'optimism'. Factor 5 includes items 5, 15 and 19 and reflects 'self-awareness. Factor 6 includes items 6, 9, 11, 13, and 17 and reflects 'lack of self confidence'. Finally factor 7 includes items 8, 9, 13 and 14 and possibly reflects a 'cognitive deficit' especially concerning the interpersonal domain.

The correlation matrix (Table 3) revealed a moderate-weak relationship of the SWN-20 and its factors with the PANSS-Positive (PANSS-P), PANSS-Negative (PANSS-N), PANSS-General Psychopathology (PANSS-G), the Simpson-Angus and the Calgary scales, and no relationship to age, education and income class.

**Table 3:** Correlation among the SWN-20 total score and factor subscales with the rest of psychometric scales and demographic variables

	**SWN**	**Factor 1**	**Factor 2**	**Factor 3**	**Factor 4**	**Factor 5**	**Factor 6**	**Factor 7**
Age	0.03	0.06	0.14	0.08	-0.01	-0.07	0.15	0.06

Education	0.07	-0.11	-0.08	-0.04	0.17	0.19	-0.13	-0.02

Income	-0.09	-0.03	-0.12	0.04	-0.04	-0.11	-0.01	-0.03

PANSS-Positive subscale	-0.19	**-0.28**	-0.17	-0.11	-0.14	-0.03	**-0.28**	**-0.33**

PANSS-Negative subscale	**-0.35**	-0.18	**-0.26**	-0.16	**-0.33**	**-0.30**	**-0.29**	-0.15

PANSS-General Psychopathology subscale	**-0.36**	**-0.29**	**-0.39**	**-0.24**	**-0.28**	-0.09	**-0.37**	**-0.31**

Calgary Depression Scale	**-0.44**	**-0.27**	**-0.37**	**-0.47**	**-0.35**	-0.07	**-0.24**	**-0.35**

Simpson-Angus Scale	**-0.22**	**-0.26**	**-0.23**	0.06	-0.12	-0.18	**-0.24**	-0.18

Values in bold are statistically significant at p < 0.05.

PANSS, Positive and Negative Symptoms Scale; SWN-20, 20-item Subjective Well-Being Under Neuroleptic Treatment Scale.

## Discussion

The Greek version of the SWN-20 is reliable with psychometric properties close to the original scale. A study similar to ours that evaluated the psychometric properties of the Italian version of the SWN showed a good performance as documented by the internal consistency, with a Cronbach α equal to 0.85 [13], very close to that of the Greek version (0.79). The study also reported a satisfactory subjective experience in the sample's patients (SWN mean total score 84.95, standard deviation (SD): 17.5) [13], whereas our findings are fairly different (SWN mean total score 66.73, SD: 14.33), probably due to the fact that our sample consisted mainly of chronic schizophrenic patients.

A German study applied structural equation modelling (SEM) to the data from 360 patients with schizophrenia in order to produce 5-item and 10-item indexes based on the SWN scale. The 5-item index produced seems to be a valid, time-saving tool for the assessment of the patients' perception of well-being, and thus quality of life [9].

Using the Quality of Life Scale (QLS) and SWN-20 scales, a prospective naturalistic study assessed the QoL and SWB of outpatients with schizophrenia on antipsychotic medication over a 12-month period. The analysis revealed the presence of four different patient groups: a group with continuously high QoL (23.2%), a group with continuously moderate QoL (48.5%), a group with low QoL (11.2%) and a group with improving QoL (19.9%) [14].

Patients and psychiatrists seem to perceive treatment and medication side effects in a very different way. A randomised double-blind multicentre trial evaluated the effects of olanzapine and clozapine on subjective well-being and clinical outcome after 26 weeks of treatment in 114 patients with schizophrenia. The results revealed only a moderate correlation between SWN and PANSS scores, indicating the difference of perception between patients and psychiatrists [15]. Our results are in accordance with these findings, showing a moderate to weak correlation between SWN and PANSS scores.

The majority of patients with schizophrenia seem to be satisfied with their life in general, although certain areas are most commonly described as dissatisfactory. Hofer *et al. *indicated partnership and mental health as the most commonly noted areas. They also concluded that SWB was negatively influenced by the depression/anxiety component of the PANSS, extrapyramidal symptoms and a negative attitude towards antipsychotics [16]. Side effects of antipsychotic medication are generally considered as a major source of subjective discomfort among patients, leading to poor SWB [17]. A study of 161 patients suffering from schizophrenia found that patients with side effects were less satisfied with life domains of subjective feelings and general activities than asymptomatic patients, and that QoL seems to be influenced by the patient's subjective response to side effects [18]. Patients receiving SGAs report a high perceived quality of life in various aspects of life, although metabolic disturbances seem to have a significant detrimental effect [19]. However, schizophrenic patients that, where switched from an SGA to a first-generation antipsychotic (FGA) for clinical reasons, reported no disadvantage concerning symptoms and quality of life over a 1-year period [20]. Moreover, a study of 1,462 patients with schizophrenia, treated either with FGAs or SGAs showed that both quality of life and symptom severity improved over the study period, regardless of the antipsychotic taken, indicating that the type of antipsychotic does not seem to have an effect on satisfaction with life [21]. Tempier *et al. *found that patients receiving SGAs had lower scores in certain items about social relationships than patients receiving FGAs, which may be at least partially attributed to the fact that patients receiving SGAs have greater expectations from life [22].

Research data also suggest that SWB is a major determinant of adherence to treatment. In a multicentre observational study of 2960 patients suffering from schizophrenia, SWB was assessed over a 12-month period, with the use of SWN-20. The results showed that that the odds for being compliant were 1.363 times higher if the SWN-20 score increased by 20 points, indicating a strong association between SWB and adherence to treatment [23]. Although most of the studies are inconclusive and their results inconsistent, SGAs seem to be superior to FGAs in ameliorating subjective tolerability and quality of life, thus improving adherence to treatment [24]. However, a study of 106 schizophrenic and bipolar patients that investigated the correlation between SWB and adherence to treatment, showed that although patients receiving SGAs reported a better subjective response than those receiving FGAs, adherence to treatment did not differ between the two groups [25]. In any case, the assessment of the patient's subjective experience may be of use in the evaluation of the differential effects of antipsychotics and their dose in SWB and thus adherence to treatment [26].

Subjective well-being may also be an index of symptomatic remission over time and thus of predictive validity for the course of the disease. A prospective study of 110 patients suffering from first episode schizophrenia or related disorders investigated the impact of early improvement of subjective experience and early improvement of rater-assessed symptoms on symptomatic remission over a 5-year period. Patients with enduring symptomatic remission had a higher mean improvement of SWB during early treatment, as assessed with the SWN-20, than those without enduring symptomatic remission, indicating an association between SWB and long-term remission [27].

In conclusion, subjective well-being is a very important, yet neglected, concept concerning the treatment of patients with schizophrenia receiving antipsychotics. The subjective effects of antipsychotic medication seem to significantly affect the patients' quality of life and willingness to stay under treatment, thus should be considered more thoroughly, both in clinical research and clinical practice. The SWN-20 is a simple, easy to use, self-report scale for the reliable assessment of the well-being of patients under treatment with neuroleptics. The Greek translation of the SWN-20 is reliable with psychometric properties close to the original scale, and can be of use in implementing the treatment of patients with schizophrenia.

## Competing interests

KNF is member of the International Consultation Board of Wyeth for desvenlafaxine and has received honoraria for lectures from AstraZeneca, Janssen-Cilag, Eli-Lilly and research grants from AstraZeneca and Pfizer Foundation

MS, SM, KA, AC and SD received support to participate in congresses by the following companies: AstraZeneca, Bristol-Myers-Squibb, Eli-Lilly, Janssen-Cilag, Lundbeck, Novartis, Organon, Pfizer, Sanofi.

## Authors' contributions

KNF designed the study and participated in the analysis of the data, interpretation and writing of the manuscript MS, KM, EP, SM, IC, KA, AC and SD participated in the gathering of the data, interpretation of the results and writing of the manuscript.
